# Dental characteristics associated with methamphetamine use: analysis using forensic autopsy data

**DOI:** 10.1186/s12903-022-02182-6

**Published:** 2022-04-26

**Authors:** Satomi Mizuno, Sachiko Ono, Ayumi Takano, Hideo Yasunaga, Hirotaro Iwase

**Affiliations:** 1 Department of Forensic Medicine, Graduate School of Medicine, The University of Tokyo, 7-3-1, Hongo, Bunkyo-ku, Tokyo, 113-0033 Japan; 2 Department of Eat-loss Medicine, Graduate School of Medicine, The University of Tokyo, 7-3-1, Hongo, Bunkyo-ku, Tokyo, 113-0033 Japan; 3 Department of Mental Health and Psychiatric Nursing, Tokyo Medical and Dental University, 1-5-45 Yushima, Bunkyo-Ku, Tokyo, 113-8510 Japan; 4 Department of Clinical Epidemiology and Health Economics, School of Public Health, The University of Tokyo, 7-3-1, Hongo, Bunkyo-ku, Tokyo, 113-0033 Japan; 5 Department of Legal Medicine, Graduate School of Medicine, Chiba University, 1-8-1 Inohana, Chuo-ku, Chiba, Chiba Prefecture 260-8670 Japan

**Keywords:** Oral hygiene, Periodontal disease(s)/periodontitis, Computed tomography, Caries, Public health, Risk factor(s)

## Abstract

**Background:**

Little is known regarding the oral conditions in nonelderly methamphetamine users, such as the presence of dental caries and periodontitis. We aimed the oral conditions between methamphetamine users and non-users stratified by age groups.

**Methods:**

In this cross-sectional study, computed tomography images were obtained from 3,338 decedents at two forensic medicine departments in Japan. Decedents aged > 20 or ≤ 64 years were included in the study and categorised into methamphetamine-detected (MA) and undetected (control) groups based on toxicological examinations. Decedents in the MA and control groups were matched for age and sex in a 1:4 ratio. The matched pairs were further categorised into young adults (20–44 years) and middle-aged adults (45–64 years). Oral characteristics, including the decayed, missing, and filled teeth (DMFT) index; periodontitis; distributional patterns of each tooth condition; and occlusal status, were compared between the MA and control groups for each age category. Among 3,338 decedents, 37 young and 55 middle-aged adults in the MA group were matched with 148 and 220 adults in the control group, respectively.

**Results:**

In the young adult group, methamphetamine use was significantly associated with higher DMFT index (mean [standard deviation], 14.2 [7.6] vs 11.0 [6.1]; *p* = 0.007), smaller number of filled teeth (2.8 [2.9] vs 5.3 [4.0]; *p* = 0.001), higher proportion of periodontitis (39.0% vs 6.8%; *p* < 0.001), and lower proportion of occlusal support (54.1% vs 81.1%; *p* = 0.001). Young adult methamphetamine users tended to have untreated decayed canines and molars and missing molars. These findings were similar to those in middle-aged adults except the tendency to have missing maxillary incisors.

**Conclusions:**

Nonelderly methamphetamine users had distinctive oral characteristics that may help screen for methamphetamine abuse through dental examinations.

**Supplementary Information:**

The online version contains supplementary material available at 10.1186/s12903-022-02182-6.

## Background

Methamphetamine causes long-lasting adverse reactions—including psychotic symptoms, addiction, anti-social behaviour, and sudden death—in exchange for euphoria [1–3]. Methamphetamine abuse has spread worldwide in the last decade [4]. In Japan, approximately 10,000 individuals are arrested for methamphetamine use every year [5]. Reportedly, methamphetamine abuse commonly starts at an age as young as 16–26 years and tends to continue into later life because of intense cravings and increasing doses required to experience euphoria [2, 6–9].

Among the distinctive characteristics of methamphetamine users is poor oral health, including severely decayed teeth and periodontitis [10–15]. However, most studies did not consider the ageing effect when describing the oral characteristics of methamphetamine users. Two studies reported that the average decayed, missing, and filled teeth (DMFT) index and proportion of periodontitis were higher in methamphetamine users than in the general population [7, 13]. These studies treated age as a confounding factor and adjusted for it; thus, the age-stratified oral characteristics of methamphetamine users remain unclear. Furthermore, these studies had potential limited-participants-derived biases, and methamphetamine usage may have been underreported because of the social stigma and mental or cognitive impairment in people with methamphetamine use disorder.

Understanding the age-stratified oral characteristics of methamphetamine users may help identify methamphetamine abuse in the general population through dental examinations. We aimed to illustrate the oral characteristics of Japanese methamphetamine users using forensic autopsy reports and post-mortem computed tomography (CT) images. We compared the number of decayed teeth, prevalence of periodontitis, and occlusal status between methamphetamine users and non-users stratified by age groups.

## Methods

### Data

This study was approved by the Ethics Committee of The University of Tokyo in Japan (10835-1) and did not require the approval of kin. Furthermore, the requirement of informed consent was waived by the ethics committee.

We collected CT reconstruction images and forensic autopsy data of 3,338 decedents from the forensic medicine departments of the University of Tokyo and Chiba University between 1 April 2013 and 17 January 2020. We performed whole-body CT and autopsies on the decedents with deaths related to criminality, unnatural causes, or unidentifiable causes of death [16]. CT scans, forensic autopsy procedures, and data collection were standardised. The autopsy data included sex, toxicological examination results, anthropometric measures, and haemoglobin A1c (HbA1c) test results. Additionally, police investigators provided the following information: name, age, medical history, and criminal record.

### Subject selection

The exclusion criteria were unidentified decedents, death > 2 weeks prior to the study; non-Japanese nationality; age < 20 years or > 65 years; missing or broken mandibular/maxilla; and selegiline/lisdexamfetamine prescriptions. We excluded individuals deceased for > 2 weeks from autopsy as blood or urine samples for toxicological examination could not be collected from skeletonised, mummified, or decomposed bodies. Non-Japanese decedents were excluded because of unknown criminal history, different regulations for illegal drugs, and different cultural backgrounds. Decedents aged < 20 years were excluded because information on criminal charges based on the Juvenile Law was unobtainable. Decedents with a missing or broken mandibula/maxilla due to severe fractures or burns were also excluded because the examination of their oral condition was impossible. Additionally, we excluded those with selegiline/lisdexamfetamine prescriptions to avoid contamination of methamphetamine/amphetamine (MA) and control groups because these drugs are metabolised into MA [17, 18].

The decedents were then classified into MA and control groups based on the toxicological examination of methamphetamine using the 3200 QTRAP ®LC–MS/MS (AB SCIEX, Massachusetts, US) and Prominence UFLC (Shimadzu Corporation, Kyoto, Japan) systems [19]. Methamphetamine is detectable in the urine for 1 week and in the plasma for 1–2 days after intake [1]. Therefore, decedents with histories of violating the stimulant control law (SCL)—restricting methamphetamine possession and use—were excluded from the control group to avoid potential contamination of the groups. After matching age and sex, we further divided the matched pairs into the young adult (20–44 years) and middle-aged (45–64 years) groups to make the number of decedents as close to equal as possible; we then compared the oral characteristics between the MA and control groups for each age category.

### Variables

We compared the background and oral characteristics of the MA and control groups for each age category. The variables included body mass index (BMI), HbA1c level, and concurrent use of other psychotropic drugs (i.e. phencyclidine, benzodiazepine, cocaine, tetrahydrocannabinol, opioid, barbiturate, and tricyclic antidepressants). BMI and HbA1c levels were measured because these variables may affect oral health [20–24]. HbA1c level was measured using blood or urine samples with the HemoCue HbA1c 501 Analyzer (Radiometer, Copenhagen, Denmark); the HbA1c level of decedents without sufficient blood samples was recorded as missing values. The decedents’ vocation was investigated as a factor to assess potential socioeconomic confounders. The concurrent use of other psychotropic drugs was examined using the Triage DOA+ (Alere Inc., Massachusetts, US) [25], simultaneously detecting commonly abused drug groups (phencyclidine, benzodiazepines, cocaine, amphetamine, tetrahydrocannabinol, opioid, barbiturate, tricyclic antidepressants, and their main metabolites).

The oral characteristics included the DMFT index; periodontitis; occlusal status; and distributional patterns in each unsound tooth category (decayed teeth, retained roots, missing teeth, filled teeth, and filled teeth with root canal treatment [RCT]). These variables were selected based on previous studies focusing on social and oral characteristics of illicit drug users, including methamphetamine users [1, 3, 26, 27]. To assess the oral condition, a dentist with 10 years of clinical experience examined the CT reconstruction images (Synapse Vincent; Fujifilm; Tokyo, Japan). The examination was blinded, and the dentist did not know which decedents were methamphetamine users. CT images provided information allowing for the detection of fractures, number of teeth, dental restoration, severe caries, and alveolar bone absorption, regardless of the decedent status (including burned and decomposing decedents) [28–30]. We calculated the DMFT index to assess the number of unsound teeth (among 28 teeth, excluding 3 molars) using CT images. The unsound teeth were then divided into five categories (decayed teeth, retained roots, missing teeth, filled teeth, and teeth filled with RCT) commonly used for personal identification in decedents [28–30]. Additionally, we described the distributional patterns of each tooth condition in the MA and control groups. The details and examples regarding the unsound tooth categories of the CT reconstructed images are shown in Additional file 1.

The length between the cementoenamel junction and alveolar crest (CEJ-AC) was used to assess the periodontal status using CT reconstruction images [31–33]. We defined severe periodontitis as CEJ-AC of ≥ 7.1 mm, i.e. the sum of the diagnostic criterion for severe periodontitis with > 6 mm of attachment loss [33], and a connective tissue attachment length of 1.1 mm [34]. If the CEJ-AC was unmeasurable owing to metal artefacts or missing teeth, it was recorded as a missing value.

We also examined the occlusal status because severe dental caries and periodontitis cause loss of vertical occlusal support. The Eichner classification system, commonly used in clinical settings, was used to assess the occlusal status by examining the patterns of missing teeth in the maxillary and mandibular dental arch [35]. We examined the maxillary and mandibular premolars and molars that maintained vertical occlusion to determine the number of occlusal support zones (0–4) and divided the decedents into three categories according to the number of occlusal support zones.

Additionally, we compared the proportion of decedents with criminal records unrelated to the SCL (non-SCL crime)—including robbery, violent crimes, theft, and physical and sexual assault—between the groups to determine whether prisons and jails (in addition to dental offices) may be settings for methamphetamine screening. In the MA group, we further investigated the history of arrests for SCL-related crimes and the duration of methamphetamine abuse to explore whether there may have been an opportunity to intervene while these individuals were still alive. We defined the duration of methamphetamine abuse as the duration between the first registered violation of the SCL and the time of death. We complied with the STROBE guidelines/checklist.

### Statistical analysis

Since the distribution of age and sex between the groups likely distorted the relationship between methamphetamine use and dental diseases, decedents in the MA group were matched with the control group for age and sex at a 1:4 ratio with replacement. We then divided matched pairs into two age categories (i.e. young and middle-aged adults). After matching, paired *t*-test for continuous variables and McNemar's test for categorical variables were performed to compare the characteristics between the MA and control groups in each age category. Differences were considered statistically significant at *p* < 0.05. We also investigated the proportion of decedents in the MA group with a history of arrests for SCL-related crimes and the duration of methamphetamine abuse. Furthermore, we performed multivariable conditional logistic regression to evaluate the association of each oral characteristic with methamphetamine use. We selected the independent variables based on the results of the univariate analyses and availability in clinical settings. The analyses were conducted separately for young and middle-aged adults. To check multicollinearity, we calculated variance inflation factors. Variance inflation factors < 10 were considered to indicate no multicollinearity. All statistical analyses were performed using R Statistical Software (version 4.0.0; R Foundation for Statistical Computing, Vienna, Austria).

## Results

**Fig. 1:**
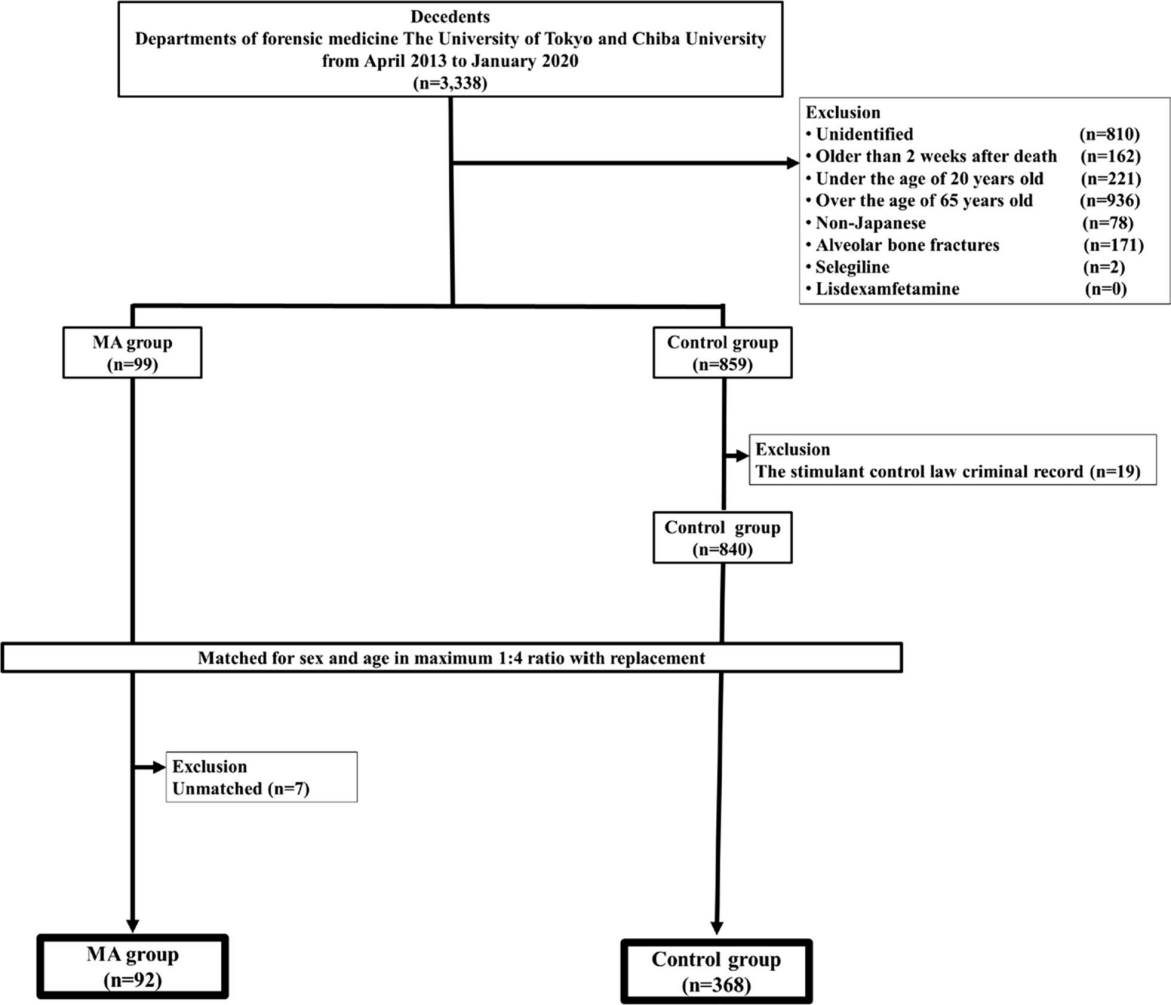
Flowchart of decedent selection and matching process. *Abbreviation MA* methamphetamine/amphetamine

We collected the data of 3,338 decedents and conducted CT, forensic autopsies, and toxicological examinations (Fig. 1). We excluded unidentified decedents (n = 810), those with a non-Japanese nationality (n = 162), those deceased for > 2 weeks before the study (n = 221), those aged < 20 years (n = 936) or > 65 years (n = 78), those with alveolar bone fractures (n = 171), those with selegiline prescription (n = 2), and those with lisdexamfetamine prescription (n = 0). The remaining 958 decedents were divided into the MA (n = 99) and control (n = 859) groups based on toxicological examination; 19 decedents with SCL-related criminal records were excluded from the control group. After the exclusion process, the number of decedents in the MA and control groups was 99 and 840, respectively. Ninety-two decedents in the MA group were matched with 368 decedents in the control group according to sex and age. Seven decedents in the MA group were excluded because they did not find matched pairs in the control group. Thus, we analysed 92 decedents (37 young and 55 middle-aged adults) in the MA group and 368 decedents (148 young and 220 middle-aged adults) in the control group.

Before matching, there was no significance in background variables in young and middle-aged adults except for age and non-SCL-criminal records (Additional file 2). After matching, the mean (standard deviation [SD]) age was 37.1 (5.9) years, and the proportion of men was 89.2% in the young adult category (Table 1). In the middle-aged category, the mean (SD) age was 52.4 (5.3) years, and the proportion of men was 85.5%. In both age categories, no significant difference was recorded regarding the vocation, BMI, HbA1c level, or the concurrent use of other psychotropic drugs between the MA and control groups, while the proportions of non-SCL-related criminal records were higher in the MA group than in the control group (45.9% vs 17.6%, *p* = 0.001 for young adults and 52.7% vs 13.6%, *p* < 0.001 for middle-aged adults).

**Table 1:** Demographic characteristics of decedents by each age category after matching

	Young adult			Middle-aged		
	MA (n = 37)	Control (n = 148)	*p*-value	MA (n = 55)	Control (n = 220)	*p*-value
Men (%)	33 (89.2)	132 (89.2)	1.00	47 (85.5)	188 (85.5)	1.00
Age (years, mean [SD])	37.1 (5.9)	37.1 (5.8)	1.00	52.4 (5.3)	52.4 (5.3)	1.00
Vocation	17 (45.9)	77 (52.0)	0.633	20 (36.4)	109 (49.5)	0.109
BMI (kg/m^2^, mean [SD])	24.5 (5.7)	22.8 (4.7)	0.07	23.1 (4.5)	22.6 (4.7)	0.525
HbA1c (%, mean [SD])	5.5 (0.5)	5.5 (1.1)	0.973	6.0 (1.5)	5.6 (1.1)	0.067
Concurrent use of drugs (%)						
Phencyclidine	0 (0)	1 (0.7)	1.00	0 (0)	0 (0)	
Benzodiazepine	3 (8.1)	5 (3.4)	0.416	3 (5.5)	23 (10.5)	0.381
Cocaine	0 (0)	0 (0)		0 (0)	0 (0)	
Tetrahydrocannabinol	2 (5.4)	6 (4.1)	1.00	1 (1.8)	3 (1.4)	1.00
Opioid	0 (0)	7 (4.7)	0.3861	1 (1.8)	3 (1.4)	1.00
Barbiturate	1 (2.7)	10 (6.8)	0.5861	1 (1.8)	9 (4.1)	0.687
Tricyclic antidepressants	2 (5.4)	0 (0)	0.051	0 (0)	4 (1.8)	0.706
Non-SCL-criminal record (%)	17 (45.9)	26 (17.6)	**0.001**	29 (52.7)	30 (13.6)	**< 0.001**

Bold values indicate that the statistical significance at *p* < 0.05

*BMI* body mass index, *HbA1c* haemoglobin A1c, *MA* methamphetamine/amphetamine, *SD* standard deviation, *SCL* stimulant control law

In the young adult category, the DMFT index was significantly higher in the MA group than in the control group (14.2 [7.6] vs 11.0 [6.1]; *p* = 0.007). In particular, the numbers of decayed teeth (3.2 [2.9] vs 1.2 [2.2]; *p* < 0.001), retained roots (1.5 [2.1] vs 0.7 [1.8]; *p* = 0.01), and missing teeth (3.7 [4.7] vs 1.8 [3.4]; *p* = 0.006) were higher in the MA group, while the number of filled teeth was significantly lower in the MA group than in the control group (2.8 [2.9] vs 5.3 [4.0]; *p* = 0.001; Table 2). The findings were similar in the middle-aged category excluding an insignificant difference in the retained roots between the MA and control groups (2.0 [3.0] vs 1.4 [3.0]; *p* = 0.169).

**Table 2:** Unsound tooth condition, periodontal status, and occlusal status in MA and control groups by age

	Young adult			Middle-aged		
	MA (n = 37)	Control (n = 148)	*p* value	MA (n = 55)	Control (n = 220)	*p* value
DMFT (Mean [SD])	14.2 (7.6)	11.0 (6.1)	**0.007**	19.3 (7.1)	16.5 (6.9)	**0.007**
Decayed teeth	3.2 (2.9)	1.2 (2.2)	**< 0.001**	2.5 (2.8)	0.9 (1.6)	**< 0.001**
Retained roots	1.5 (2.1)	0.7 (1.8)	**0.01**	2.0 (3.0)	1.4 (3.0)	0.169
Missing teeth	3.7 (4.7)	1.8 (3.4)	**0.006**	9.5 (7.8)	6.0 (7.3)	**0.002**
Filled teeth	2.8 (2.9)	5.3 (4.0)	**0.001**	2.6 (3.5)	4.5 (4.3)	**0.001**
Teeth filled with RCT	2.8 (3.7)	2.1 (3.1)	0.195	2.8 (3.7)	3.8 (3.9)	0.107
Periodontitis (%)	14 (39.0)	10 (6.8)	**< 0.001**	30 (60.0)	25 (13.4)	**< 0.001**
Eichner's classification (%)						
A: Full support	20 (54.1)	120 (81.1)	**0.001**	14 (25.5)	120 (54.5)	**< 0.001**
B: 1–3 molar support and 0 molar support with anterior support	15 (40.5)	26 (17.6)	**0.005**	26 (47.3)	79 (35.9)	0.163
C: No support	2 (5.4)	2 (1.4)	0.376	15 (27.3)	21 (9.5)	**0.001**

Bold values indicate that the statistical significance at *p* < 0.05

*MA* methamphetamine/amphetamine, *DMFT* the decayed, missing, and filled teeth index, *RCT* root canal treatment, *SD* standard deviation

The proportion of decedents with periodontitis was higher in the MA group than in the control group for the young adult category (39.0% vs 6.8%, *p* < 0.001). Although not significant, the MA group for the middle-aged category had a higher proportion of decedents with periodontitis than the control group, though this difference was not significant.

**Fig. 2:**
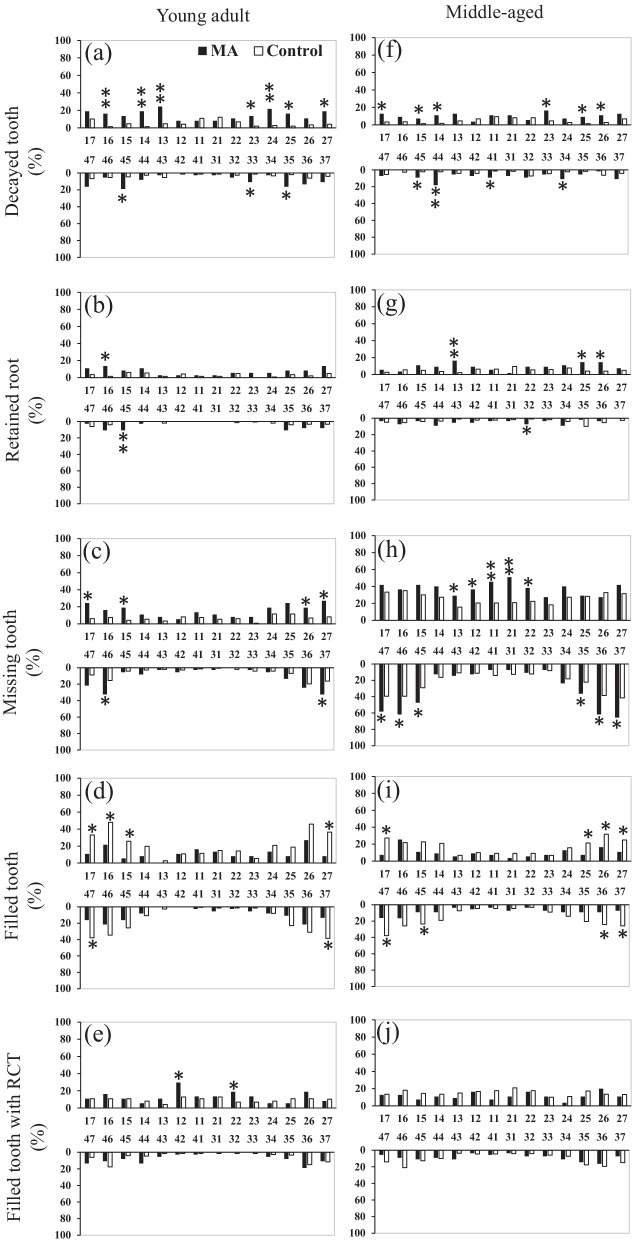
Distribution pattern by each unsound tooth category. *Abbreviations MA* methamphetamine/amphetamine, *RCT* root canal treatment. *p < 0.05, ^⁑^*p* < 0.001

The distributional patterns of each unsound tooth category are shown in Fig. 2. In the young adult category, there were more decayed and retained roots and missing maxillary and mandibular molars in the MA group than in the control group (Fig. 2a–c). Decayed maxillary canines, maxillary premolars, and maxillary molars were significantly associated with methamphetamine use (Fig. 2a). Conversely, significantly fewer filled molars were observed in the MA group than in the control group (Fig. 2d). Additionally, the MA group had more maxillary incisors filled with RCTs than the control group (Fig. 2e). In the middle-aged category, the number of missing maxillary incisors and mandibular molars was higher in the MA group than in the control group (Fig. 2h), while the remaining findings were similar to those in the young adult category (Figs. 2f, g, i).

Regarding Eichner's classification, the proportion of decedents with full occlusal support was lower in the MA group (54.1% vs 81.1%; *p* = 0.001 for young adults and 25.5% vs 54.5%, *p* < 0.001 for middle-aged adults) (Table 2).

The proportion of methamphetamine users with histories of SCL crime and the median (interquartile range) years of methamphetamine abuse were 65.9% and 4 (2.0–8.8) years, respectively, in young adults and 70.7% and 5 (2–24.5) years, respectively, in middle-aged adults (data not shown).

Additional file 3 shows the results of multivariable conditional logistic regression analysis for young and middle-aged adults. The model included methamphetamine use as a dependent variable and the number of decayed teeth, periodontitis, and whether descendants had full occlusal supports as independent variables based on univariate analysis results. We confirmed that all variance inflation factors were < 10 in the multivariable logistic regression model. In the young adult category, the number of decayed teeth (odds ratio [OR] 1.4; 95% confidence interval [CI] 1.2–1.7) and periodontitis (OR 10.3; 95% CI 3.4–33.3) were significantly associated with methamphetamine use. In middle-aged methamphetamine users, the number of decayed teeth (OR 1.2; 95% CI 1.0–1.4), periodontitis (OR 7.2; 95% CI 3.4–15.4), and full occlusal support (OR 0.4; 95% CI 0.2–0.8) were significantly associated with methamphetamine use.

## Discussion

Our study investigated the oral characteristics of methamphetamine users in Japanese nonelderly—young and middle-aged adults—people who died of unnatural causes using forensic autopsy images and reports. The number of untreated teeth and proportion of decedents with severe periodontitis were higher, and the proportion of decedents with full occlusal support zones was lower in methamphetamine users than in non-users. Although most findings were similar across the age categories, missing maxillary incisors and mandibular molars were distinctive among middle-aged methamphetamine users.

The proportion of the decedents with untreated decayed teeth and severe periodontitis was higher in methamphetamine users in both age categories. The results support preliminary findings of previous studies lacking generalisability and confounding adjustment [7, 15]. The mechanism of poor oral health in methamphetamine users may be methamphetamine-induced central inhibition causing xerostomia, hyperactive bruxism, and neglect of oral hygiene [7, 36]. Moreover, severe tooth problems in methamphetamine users tend to be left untreated because of cognitive impairment, depression, financial problems, and the stigma associated with illicit drug use [11]. Our results showed that methamphetamine users had more untreated tooth decay, missing teeth, and severe periodontitis than non-users regardless of the age category.

Different distribution patterns of missing teeth were recorded between the young adult and middle-aged methamphetamine users. More missing maxillary incisors and mandibular molars were observed in middle-aged methamphetamine users than in non-users, confirming the findings of previous studies [13]. In contrast, the association between methamphetamine use and missing maxillary incisors was insignificant in young adult methamphetamine users. One previous study inferred that the missing anterior teeth resulted from severe rampant caries caused by xerostomia [13]. Another explanation for anterior missing teeth in middle-aged adults is that the gradual loss of posterior occlusal supports due to caries or periodontitis at a younger age may cause chronic occlusal trauma of the front teeth later in life [37, 38]. Our results showed that methamphetamine users had fewer molar supporting zones than non-users in their youth. However, we could not confirm whether the loss of occlusal support at an early age affected the later loss of anterior teeth because of the cross-sectional design of the study.

Our results showed that oral characteristics, including the number of untreated decayed teeth and missing teeth, severe periodontitis, and few occlusal supports, were distinctive in methamphetamine users. This information could be used as a simple and non-invasive screening method for methamphetamine use disorder. Dental check-ups, preoperative assessment for anaesthesia, tracheal intubation, and oral care management in hospitals, wards, or jails are candidate settings for screening. The medical staff and correctional officers should be aware of the possibility of methamphetamine abuse when encountering patients with many untreated decayed teeth and severe periodontitis for their age. In young adults, this is particularly true if they present with a loss of occlusal support due to dental treatment interruptions, severe caries, or periodontitis. This information enables screening for methamphetamine abuse and adequate dental/medical management for patients under the influence of methamphetamine. Furthermore, these clinical settings or correctional institutions may be a platform to distribute information on interventional programmes to people with suspected methamphetamine addiction [21]. Suspected methamphetamine users should be notified that their information is used to help them recover and not report them to the authorities. In addition to oral assessment, saliva for methamphetamine use screening can be collected during oral examination [39].

Understanding the oral condition of methamphetamine users can also contribute to human identification. With reference to criminal records, distinctive oral characteristics may help in the identification of bodies. In contrast, poor oral conditions caused by methamphetamine abuse may make the identification process more difficult even with prior dental information. Future studies should investigate the extent to which methamphetamine abuse affects the human identification process.

This study has several limitations. First, the study might lack generalisability because the data were obtained from the forensic autopsy of the decedents. Second, we only investigated the known or reported oral factors associated with methamphetamine use; further research is warranted to examine other factors associated with methamphetamine use, such as socioeconomic status. However, we simultaneously investigated more variables than the previous studies, including the occlusal status with adjustments for sex and age. Third, the dental condition determined through CT reconstructed images was inaccurate compared with those determined through orthopantomography or intraoral X-ray images because of metal artefacts caused by dental materials. We may have failed to detect small caries and composite resin fillings in the approximal surfaces of teeth. Fourth, the study cannot exclude potential misclassification of the MA and control groups. A toxicological examination can detect methamphetamine use approximately 1 week after its intake; therefore, we may have misclassified occasional methamphetamine users into the control group. However, the typical dosing pattern of methamphetamine abuse is four doses/day to avoid withdrawal symptoms such as depression, anxiety, suicidal ideation, and panic [1]. Therefore, we believe that most individuals with typical methamphetamine use disorder were classified into the MA group.

## Conclusions

We illustrated the dental characteristics of methamphetamine use in nonelderly Japanese individuals who died of unnatural causes. The oral health characteristics were poorer in methamphetamine users than in non-users regardless of age categories, excluding a few minor differences. Thus, the information obtained in this study could be used to screen for nonelderly methamphetamine users in clinical settings.

## Supplementary Information

**Additional file 1.** Examples and two-dimensional computed tomography (CT) images (axial, sagittal, and coronal plane images) of the unsound tooth categories. Description of data: Examples and two-dimensional CT images.

**Additional file 2.** Demographic characteristics of decedents by each age category before matching. Description of data: Demographic characteristics of decedents by each age category before matching.

**Additional file 3.** Results of multivariable conditional logistic regressions analysis of three factors associated with methamphetamine use. Description of data: Results of multivariable conditional logistic regressions analysis of three factors associated with methamphetamine use.

## Data Availability

The datasets generated and/or analysed during the current study are not publicly available due ethical and legal restrictions associated forensic cases but are available from the corresponding author on reasonable request.

## Abbreviations

BMI: Body mass index
CT: Computed tomography
DMFT: Decayed, missing, and filled teeth
MA: Methamphetamine/amphetamine
RCT: Root canal treatment
SCL: Stimulant control law
SD: Standard deviation
