# Supplementary material for: Dental characteristics associated with methamphetamine use: analysis using forensic autopsy data
Source: BMC Oral Health. 2022 Apr 26;22:141. doi: 10.1186/s12903-022-02182-6 (PMC9044830; doi:10.1186/s12903-022-02182-6)
Supplement: Supplementary file 1 — Additional file 1. Examples and two-dimensional computed tomography (CT) images (axial, sagittal, and coronal plane images) of the unsound tooth categories. Description of data: Examples and two-dimensional CT images. [file 12903_2022_2182_MOESM1_ESM.docx]

**Additional File 1.** Examples and two-dimensional computed tomography (CT) images (axial, sagittal, and coronal plane images) of the unsound tooth categories


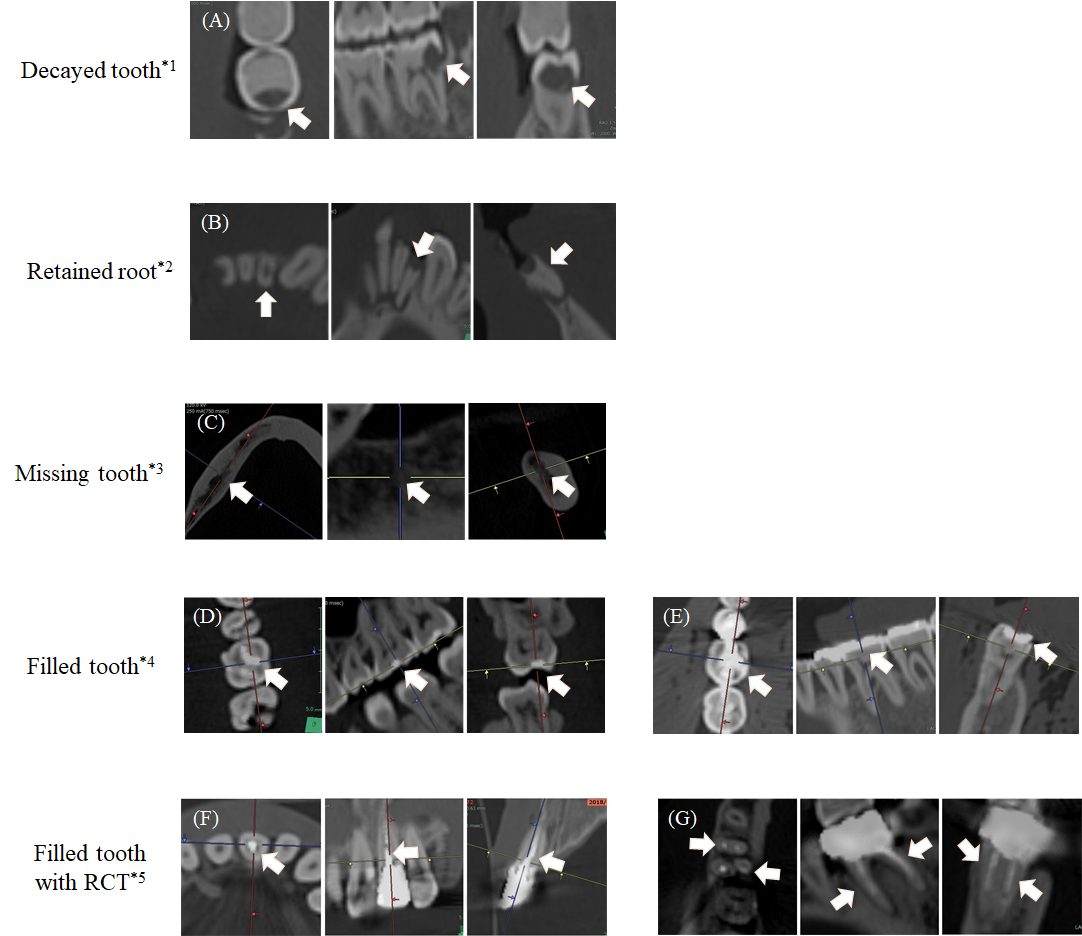


Abbreviation: RCT, root canal treatment

Left: axial plane two-dimensional CT images; Central: sagittal plane two-dimensional CT images, right: coronal plane two-dimensional CT images

*1: Decayed teeth are defined as teeth with defective regions reaching the dentin or pulp.

*2: Retained roots are defined as teeth observed with only the root embedded in the maxilla/mandible.

*3: Missing teeth are defined as teeth unobserved in the maxilla/mandible.

*4: Filled teeth are defined as teeth with dental materials and non-root canal fillings.

*5: Teeth filled with RCTs are defined as teeth with dental materials and root canal fillings. In clinical practice, RCTs are conducted for severe caries reaching the dental pulp.

(A) Tooth with composite resin; (B) Tooth with metal inlay; (C) Tooth with RCT setting the resin faced cast crown; (D) Tooth with RCT setting the full metal cast crown; (E) Tooth with caries reaching the dentin and pulp; (F) The root, the only remaining part of the tooth, seen embedded in the mandible; (G) Unobserved teeth.
