# Supplementary material for: Dental characteristics associated with methamphetamine use: analysis using forensic autopsy data
Source: BMC Oral Health. 2022 Apr 26;22:141. doi: 10.1186/s12903-022-02182-6 (PMC9044830; doi:10.1186/s12903-022-02182-6)
Supplement: Supplementary file 2 — Additional file 2. Demographic characteristics of decedents by each age category before matching. Description of data: Demographic characteristics of decedents by each age category before matching. [file 12903_2022_2182_MOESM2_ESM.docx]

**Additional File 2.** Demographic characteristics of decedents by each age category before matching

|  | Young adult | | |  | Middle aged | | |
| --- | --- | --- | --- | --- | --- | --- | --- |
|  | MA | Control | p-value |  | MA | Control | p-value |
|  | (n=41) | (n=348) |  |  | (n=58) | (n=492) |  |
| Men (%) | 33 (80.5) | 240 (69.0) | 0.179 |  | 47 (81.0) | 391 (79.5) | 0.915 |
| Age (years, mean [SD]) | 36.95 (5.85) | 32.99 (7.22) | 0.001 |  | 52.69 (5.39) | 54.40 (5.92) | 0.036 |
| Vocation | 18 (43.9) | 172 (49.4) | 0.614 |  | 21 (36.2) | 223 (45.3) | 0.237 |
| BMI (kg/m^2^, mean [SD]) | 24.16 (5.47) | 22.38 (4.79) | 0.027 |  | 22.83 (4.56) | 22.39 (4.68) | 0.498 |
| HbA1c (%, mean [SD]) | 5.50 (0.52) | 5.52 (1.17) | 0.922 |  | 5.97 (1.44) | 5.80 (1.39) | 0.494 |
| Concurrent use of drugs (%) |  |  |  |  |  |  |  |
| Phencyclidine | 0 (0.0) | 1 (0.3) | 1 |  | 0 (0) | 0 (0) |  |
| Benzodiazepine | 3 (7.3) | 17 (4.9) | 0.769 |  | 4 (6.9) | 29 (5.9) | 0.994 |
| Cocaine | 0 (0.0) | 1 (0.3) | 1 |  | 0 (0) | 0 (0) |  |
| Tetrahydrocannabinol | 2 (4.9) | 10 (2.9) | 0.822 |  | 1 (1.7) | 7 (1.4) | 1 |
| Opioid | 0 (0.0) | 10 (2.9) | 0.563 |  | 1 (1.7) | 9 (1.8) | 1 |
| Barbiturate | 1 (2.4) | 18 (5.2) | 0.7 |  | 1 (1.7) | 13 (2.6) | 1 |
| Tricyclic antidepressants | 2 (4.9) | 3 (0.9) | 0.154 |  | 0 (0.0) | 11 (2.2) | 0.512 |
| Non-SCL-criminal record (%) | 17 (41.5) | 43 (12.4) | <0.001 |  | 29 (50.0) | 60 (12.2) | <0.001 |

BMI, body mass index; HbA1c, haemoglobin A1c; MA, methamphetamine/amphetamine; SD, standard deviation; SCL, stimulant control law

We used the Student’s t-test for continuous variables and the chi-squared test for categorical variables.
