# Supplementary material for: Dental characteristics associated with methamphetamine use: analysis using forensic autopsy data
Source: BMC Oral Health. 2022 Apr 26;22:141. doi: 10.1186/s12903-022-02182-6 (PMC9044830; doi:10.1186/s12903-022-02182-6)
Supplement: Supplementary file 3 — Additional file 3. Results of multivariable conditional logistic regressions analysis of three factors associated with methamphetamine use. Description of data: Results of multivariable conditional logistic regressions analysis of three factors associated with methamphetamine use. [file 12903_2022_2182_MOESM3_ESM.docx]

**Additional File 3.** Results of multivariable conditional logistic regressions analysis of three factors associated with methamphetamine use

|  | After matching | | | | | | |
| --- | --- | --- | --- | --- | --- | --- | --- |
|  | Young adult  (n=185) | | |  | Middle aged  (n=275) | | |
|  | Odds ratio | 95% CI | p-value |  | Odds ratio | 95% CI | p-value |
| Decayed teeth (mean [SD]) | 1.4 | 1.2–1.7 | <0.001 |  | 1.2 | 1.0–1.4 | 0.0179 |
| Periodontitis (%) | 10.3 | 3.4–33.3 | <0.001 |  | 7.2 | 3.4–15.4 | <0.001 |
| Eichner's classification A: All support (%) | 0.4 | 0.2–1.1 | 0.075 |  | 0.4 | 0.2–0.8 | 0.0126 |

Abbreviation: SD: standard deviation, CI: confidence interval

Akaike’s information criteria of the model in young adult and middle-aged methamphetamine users are 140.89 and 192.49, respectively.
